# Interference in speaking while hearing and vice versa

**DOI:** 10.1038/s41598-019-41752-7

**Published:** 2019-03-29

**Authors:** Raphaël Fargier, Marina Laganaro

**Affiliations:** 1 Aix-Marseille University, Institute of Language, Communication and Brain, Aix-en-Provence, France; 2 Faculty of Psychology and Educational Sciences, University of Geneva, Geneva, Switzerland

## Abstract

Even when speakers are not actively doing another task, they can be interfered in their speech planning by concurrent auditory stimuli. In this study, we used picture naming with passive hearing, or active listening, combined to high-density electroencephalographic (EEG) recordings to investigate the locus and origin of interference on speech production. Participants named pictures while ignoring (or paying attention to) auditory syllables presented at different intervals (+150 ms, +300 ms or +450 ms). Interference of passive hearing was observed at all positive stimulus onset asynchronies (SOA) including when distractors appeared 450 ms after picture onset. Analyses of ERPs and microstates revealed modulations appearing in a time-window close to verbal response onset likely relating to post-lexical planning processes. A shift of latency of the N1 auditory component for syllables displayed 450 ms after picture onset relative to hearing in isolation was also observed. Data from picture naming with active listening to auditory syllables also pointed to post-lexical interference. The present study suggests that, beyond the lexical stage, post-lexical processes can be interfered and that the reciprocal interference between utterance planning and hearing relies on attentional demand and possibly competing neural substrates.

## Introduction

Although speaking is usually done effortlessly, there is a wealth of situations in which it becomes nearly impossible to continue producing utterances with the same ease. Consider for example how difficult it is to carry on talking on the phone while supervising your child risking to fall, or even when someone is using a pneumatic drill while you are speaking. In the first situation, the speaker is in an (active) dual-task condition, which has repeatedly been shown to interfere with speech planning^1,2^. In the second situation, the speaker is not actively doing another task, but he/she is nevertheless interfered by passively hearing other auditory stimuli. Whereas interference in active dual-task conditions can be attributed to both, divided attentional resources or competing neural resources (i.e. cross-talk), in the passive hearing condition the attentional load is minimized. Here, we investigate, by combining Stimulus Onset Asynchronies (SOA) and high-density electroencephalographic (EEG) recordings, whether and when interference occurs when participants hear (but are not required to actively react upon) concurrent stimuli while they are planning speech.

The attentional requirements of cognitive tasks have been traditionally studied with dual-task paradigms^3,4^. Running several tasks at the same time usually leads to reduced accuracy and slower reaction times (RTs) compared to when the same tasks are carried out in isolation^5^. Such dual-task interference effects are assumed to reflect resource limitations or central bottleneck affecting response selection^3,5–10^.

Driving is one of the best examples of daily situations that require sharing attention over multiple tasks, such as visual processing of traffic-related objects and auditory processing (e.g. radio programs, conversation). The sensitivity of these tasks to attentional interference has been demonstrated in several studies^11^, but some^12^ further indicated that the processing costs on visual tasks only emerged when participants had to act upon the concurrent auditory information. This indicates that central bottlenecks are bound to active, high demanding tasks. Other studies explored visual-auditory tasks, notably in the context of cell phone conversations while driving^13^ and suggested that it is not the mere listening to auditory messages that impacts the resolution of tasks, nor the simple act of articulating^14^. Actually, dual-task interference was obtained when participants had to *generate* utterances^14,15^, thus suggesting the critical role of utterance planning and lexical selection in capturing attention. As utterance planning involves the efficient selection of a message one intends to convey and the planning at different linguistic levels (lexical, grammatical, phonological), some or all planning processes must require attentional control to some extent. It has been proposed that only some utterance planning processes, especially conceptual planning^16^ and lexical selection^17,18^ require attentional control, other processes, such as the encoding of the phonological form of the utterance and its execution, being more automatic.

This issue has been addressed focusing on language planning processes with gaze tracking studies or dual-tasks^1,18–25^. Yet, conflicting results are available on whether post-lexical processes (e.g. utterance form encoding) are affected in dual-task conditions^22^. Most dual-task studies investigated the impact of reacting to tones on speech planning processes therefore reflecting active interference by non-verbal concurrent auditory stimuli. Yet, interference of speech planning by auditory distractors can also be found in some experimental conditions of picture-word interference tasks^26–28^. These studies often reported larger interference when distractors were displayed with picture onset compared to delayed asynchronies. In the seminal work by Schriefers *et al*., unrelated words delayed production latencies relative to silence at SOA = 0 ms and at SOA = 150 ms (Exp2) but not at SOA = +300 ms^26^. In subsequent studies only distractors semantically or phonologically related to the target words were analysed to infer on word planning processes and usually compared to those of unrelated words, but not to silence. These results were taken to suggest that that interference arises at the lexical stage (and not later), but there are also some cues that unrelated distractors can interfere with picture naming when they are displayed beyond 150 ms (see^29^ with between-subjects SOAs). In line with the idea that the amount of interference depends on the degree of similarity between the tasks that are run concurrently^7,30–32^, recent evidence from verbal production tasks indeed suggest that a concurrent active task involving verbal stimuli has a larger interfering effect than a concurrent task involving non-verbal stimuli^2,25,33,34^. In addition to larger interference of verbal stimuli, Fargier and Laganaro (2016) showed that verbal dual-task interference was associated with Event-related potentials (ERPs) modulations in a time-window likely corresponding to post-lexical (word form or monitoring) processes. Altogether, these results suggest that whereas speakers may retrieve the phonological form of a word easily when concurrently responding to non-verbal stimuli, it takes more effort or time when listening to verbal distractors. As argued by Fargier & Laganaro (2016) these cross-talk effects can emerge at the phonological level because the neural networks used for the processing of the distractors are already engaged in the retrieval of the segmental content of the word to be uttered.

On the one hand, dual-task studies involving speaking while active listening highlight that all speech planning stages require attentional demand but for post-lexical processes this seems to be bound to specific circumstances (e.g. cross-talk between verbal stimuli/networks). On the other hand, several visual-auditory studies indicate that dual-task interference can be reduced or suppressed if the auditory task is less demanding^13–15^. In case of concurrent auditory stimuli during speech perception and comprehension (i.e. while listening, such as in the Cocktail Party conditions^35^), the listener must somehow select the particular auditory stream he/she has to attend and engage his/her attention to^36–38^. This is however not the case when *planning* for speaking. It is therefore unclear whether the interference by concurrent auditory verbal stimuli observed on planning for speaking is only due to increased attentional load and to divided attention or can also result from competing resources.

In the present study, we used a dual-task paradigm combined to high-density electroencephalographic (EEG) recordings to further explore these issues. Participants were asked to name pictures which were presented with concurrent auditory verbal stimuli (syllables) that had to be ignored (i.e. passive hearing) or detected (active listening). We also manipulated stimulus onset asynchronies (SOAs: +150 ms, +300 ms or +450 ms relative to picture onset). Given the time estimates of speech planning processes in picture naming^39^ and previous ERPs results with dual-task paradigms^25^ we expect interference at late SOAs (i.e. +300 and +450 ms), while participants are engaged in word form (phonological) planning and internal monitoring of the planned speech. Further, if interference of auditory verbal stimuli while planning word form is not purely attentional, interference should be observed also while passive listening, with the underlying assumption that passively hearing unrelated syllables attracts less attention than active listening. Participants also underwent each task (picture naming and hearing auditory syllables) in isolation, enabling the comparison of the reciprocal interference of concurrent stimuli on picture naming and on auditory processing. Microstates analyses and analyses of waveform differences were performed to determine whether processing stages underlying the resolution of the tasks are sensitive to passive interference.

## Results

### Picture naming with passive hearing of auditory syllables

#### Behavioral results

After the exclusion of subjects with bad EEG signal (N = 4), the behavioral analyses were performed on 20 subjects. For production latencies (reaction time, RT) analyses, incorrect responses and production latencies beyond 2.5 standard deviations (16.6% of the data) as well as trials excluded for bad EEG signal (8.8% of the data) were removed.

**Figure 1:**
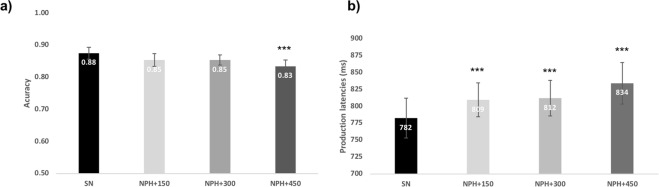
Behavioral effects of hearing concurrent auditory syllables during picture naming. Accuracy and production latencies are presented for the single condition (SN) and in naming while hearing syllables at different SOAs (NPH + 150, NPH + 300 and NPH + 450).

For statistical analyses, generalized (for binomial distribution) and linear mixed-effects regression models were performed respectively on accuracy and production latencies with participants and items as cross random effects. Condition (SN, NPH + 150, NPH + 300 and NPH + 450) and stimuli order were entered as fixed factors. A gradual interference was observed in naming while passive hearing with increasing SOA as indicated by lower accuracies and slower reaction times (see Fig. 1a,b). Significant differences in accuracy were found between the single condition (SN) and naming while passively hearing when distractors appeared at +450 ms (88% vs. 83%; z = −3.39; p < 0.001).

With regard to production latencies, a main effect of condition (F(3,4271) = 19.95, p < 0.001) and of stimuli order (F(1,4517) = 6.74, p = 0.009) were obtained. The maximal interference was found with SOA of +450 ms with mean production latencies of 52 ms slower than in the SN (t(4262) = 7.64, p < 0.001). Increased production latencies in passive interference tasks relative to single naming were also observed at SOA = +150 ms (27 ms slower than in SN, t(4276) = 3.596; p < 0.001) and at SOA = +300 ms (30 ms slower, t(4266) = 4.66; p < 0.001). Other statistical comparisons indicate that the increased production latencies are maximal at SOA = +450 ms compared to other SOA (see Appendix B).

#### ERP results

**Naming during passive hearing**: ***Waveform analysis***: Waveform analyses were performed on difference waves for stimulus-aligned epochs (NPH –PH; see Material and Methods) and on raw data for response-aligned epochs.

**Figure 2:**
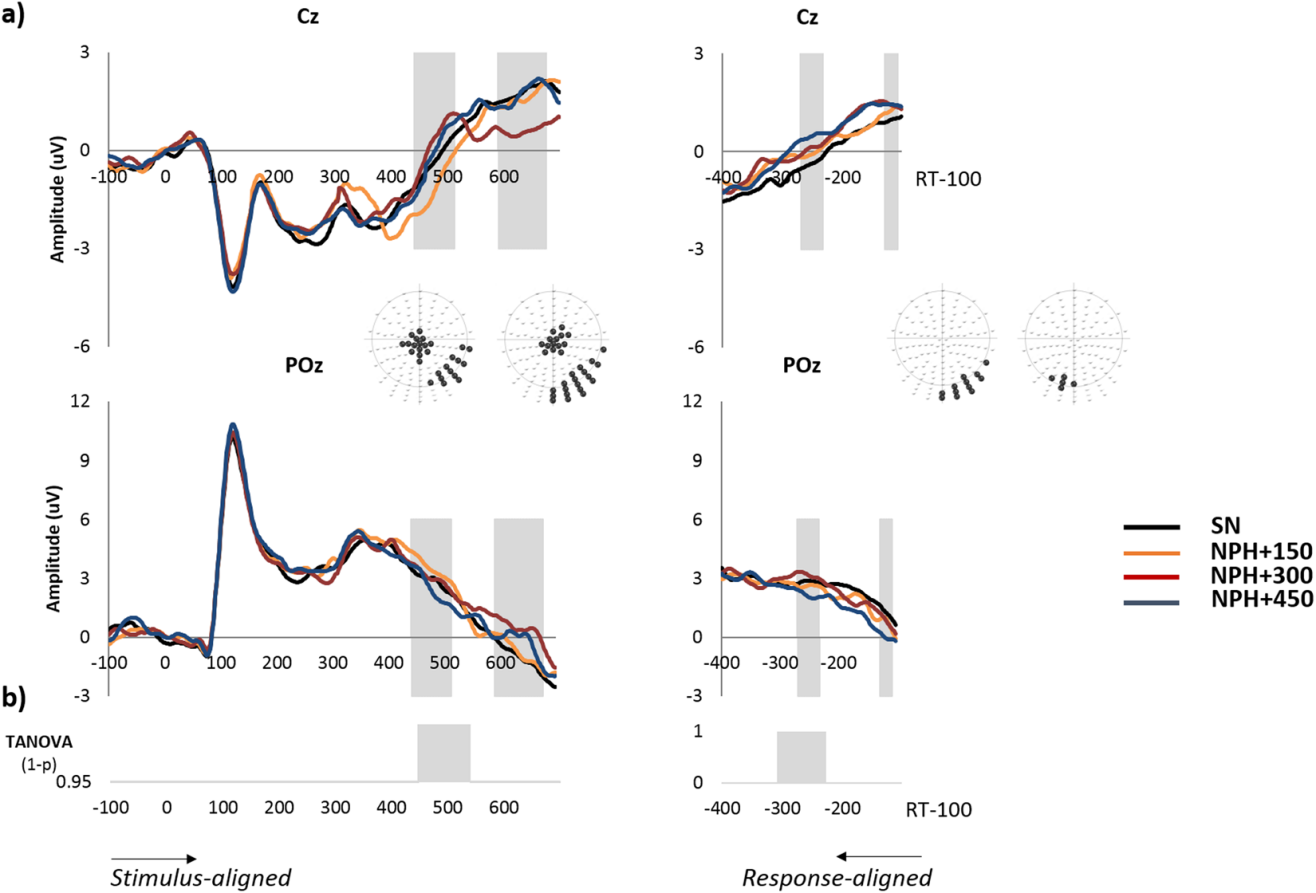
(**a**) Example of stimulus-aligned group averaged ERP waveforms for the picture naming single condition SN (black line) and waveform differences for the naming while hearing tasks (NPH-PH) at different SOAs: +150 (orange line), +300 (red line) and +450 (blue line) on electrodes Cz and POz. Raw response-aligned ERPs are also presented. The gray shaded areas represent significant differences observed with the parametric ANOVA, and the corresponding scalp distributions of the effects are provided. (**b**) Significant differences on global dissimilarity as calculated with the TANOVA are also presented.

Figure 2a shows examples of waveforms from 100 ms prior to picture onset to 700 ms afterwards on two selected electrodes (CZ, POz) and the time points of significant amplitude differences as revealed by the ANOVA that contrasted the four conditions. Consistent differences appeared on two time-windows from about 440 ms post picture onset to 514 ms and from about 595 to 675 ms after picture onset on central and posterior sites. Planned comparisons indicated that differences in amplitudes in the first period corresponded to differences between the single naming task and picture naming with passive hearing of distractor syllables presented at 150 ms, whereas the second period corresponded to differences between single picture naming and picture naming with passive hearing of distractor syllables presented at 300 ms. The TANOVA also revealed significant differences between conditions on the global dissimilarity from 450 ms to 550 ms post picture onset (see Fig. 2b).

On response-aligned epochs, significant differences on posterior sites were also found on waveform amplitudes on two short time-windows: from 270 to 234 ms prior to vocal onset and from 110 to 130 ms prior to vocal onset. The TANOVA also revealed significant differences between conditions on the global dissimilarity from 310 ms to 230 ms prior to vocal onset (see Fig. 2b).

**Figure 3:**
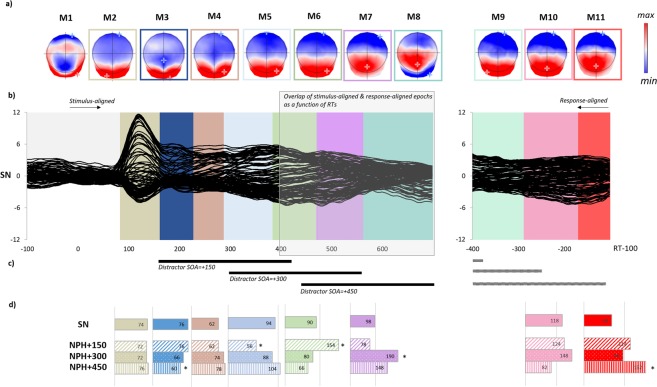
(**a,b**) Grand-average ERPs (128 electrodes) for the picture naming single task from 100 ms prior to picture onset to 100 ms before the verbal response and temporal distribution of the microstates revealed by the spatio-temporal segmentation analysis. Microstates are color-coded. The white shaded area illustrates overlap between stimulus-aligned and response-aligned data as a function of mean production latency. Map templates for the eleven microstates observed from 100 ms prior to picture onset to RT-100 are also provided with positive (red) and negative (blue) values being displayed as well as maximal and minimal scalp field potentials (**a**). (**c**) Black lines illustrate the temporal profile of the appearance of distractors when aligned to stimuli. For information, gray dotted lines illustrate the same temporal profile when aligned to the response as a function of mean production latencies (**d**) The histograms indicate the relative duration of each microstate and is given for each condition including the picture naming single condition displayed (SN) and the naming while hearing with different SOAs: NPH + 150; NPH + 300 and NPH + 450. The actual duration of the microstates is displayed. *indicate significant differences (p < 0.05) observed on any specific microstate contrasting SN with each of the passive interference conditions.

***Microstates analysis***: A spatio-temporal segmentation analysis was run separately on the grand averaged stimulus-aligned (difference waves) and the response-aligned (raw) ERPs. The analysis yielded 8 different microstates on the stimulus-aligned data and 3 microstates on the response-aligned data in all conditions but with different time-distributions (see Fig. 3). Microstate fitting statistics were computed for each naming while hearing condition with distractors presented at different SOAs (NPH + 150, NPH + 300, NPH + 450) compared to the single naming condition (SN). The duration of each microstate in each condition is presented with histograms in Fig. 3d. Relative to single picture naming, a progressive shift in the microstates yielding longer duration across conditions is observed: M6 for picture naming with passive hearing of distractor syllables presented at 150 ms relative to single picture naming (mean duration 154 ms vs. 90 ms, p < 0.001); M7 for picture naming with passive hearing of distractor syllables presented at 300 ms relative to single picture naming (mean duration 190 ms vs. 98 ms, p < 0.001) and M11 for picture naming with passive hearing of distractor syllables presented at 450 ms compared to single picture naming (mean duration 156 ms vs. 72 ms, p < 0.001). These shifts are consistent with the shift of the presentation of the auditory syllable (Fig. 3c). Results on the maps M8 and M9, which are at the upper and lower limits of the extracted ERP periods not analyzed as they reflect the consequence of different durations of previous maps in the fitting in a limited time-window.

To sum up, interference in naming while hearing compared to the single naming task seems to be associated with differences in time distributions of several microstates, with the maximal interference for picture naming with passive hearing of distractor syllables presented at 450 ms being associated with increased duration of the last microstate close to verbal onset (M11).

**Hearing while naming**: In order to compare the auditory single task (passive hearing, PH) with auditory processing while naming, we computed waveform differences between picture naming while hearing (NPH) and picture naming alone (SN) (see Method section). The comparison between auditory ERPs in hearing alone and hearing while naming was computed on the Global Field Power aligned to the onset of auditory syllables.

**Figure 4:**
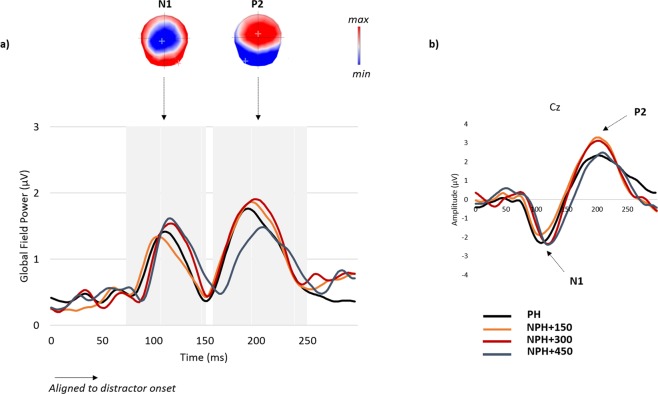
(**a**) Global Field Power of the grand averages of the raw auditory single task (PH; in black) and of the waveform differences of passive interference tasks (NPH-SN) with different SOAs (NPH + 150 in orange; NPH + 300 in red; NPH + 450 in blue). The scalp topography of the waveforms in the time-windows likely corresponding to the gray shaded areas of the N1 (between 75 and 150 ms) and P2 (between 160 and 250 ms) are provided. Positive (red) and negative (blue) values as well as maximal and minimal scalp field potentials are indicated. (**b**) An example of waveforms at the electrode Cz is displayed.

We computed the analysis on two auditory-evoked potentials: the N1 and the P2 (see Fig. 4). The latency and amplitude of the GFP maxima was extracted between 75 ms and 150 ms post stimulus onset likely corresponding to the auditory N1 component time-window (peaking around 110 ms in the present data, see Fig. 4) and between 160 and 250 ms post stimulus onset corresponding to the auditory P2 (peaking around 200 ms, see Fig. 4). Statistical analysis (Dunnett’s test) revealed significantly delayed latency of GFP maxima of the N1 only for picture naming with passive hearing of syllables presented at 450 ms, as compared to passive hearing (maxima at 126 ms for NPH + 450 vs. 110 ms for PH, p = 0.05). The latency of GFP maxima was at 110 ms and 116 ms for picture naming with passive hearing of distractor syllables presented at 150 ms and 300 ms respectively, which were not significantly different from passive hearing (respectively p = 0.99 and p = 0.8). Moreover, a significant negative correlation was observed between the latency of GFP maxima in picture naming with passive hearing of distractor syllables presented at 450 ms and production latencies (r = −0.48, p = 0.032) indicating that the faster were the subjects the more delayed was the GFP maxima in the auditory N1 time window. Significant differences in amplitudes of the N1 were observed when comparing each picture naming with passive hearing task against passive hearing (see Appendix C). No significant effect was present on P2 latency (see Appendix 2) but its amplitude was significantly reduced in passive hearing (2.4 µV) compared to picture naming with passive hearing of distractor syllables presented at 300 ms (2.98, p = 0.06).

To sum up all the results, passive hearing interference is observed on microstates computed on picture naming likely indicating modulations of speech planning processing stages but also on auditory components likely indicating modulations of auditory processing, i.e. reciprocal interference. Maximal effects were seen when auditory stimuli appeared 450 ms after picture onset.

In the following, we report similar analyses performed on data obtained from picture naming with active listening to auditory syllables.

### Picture naming with active listening to auditory syllables

#### Behavioral results

**Figure 5:**
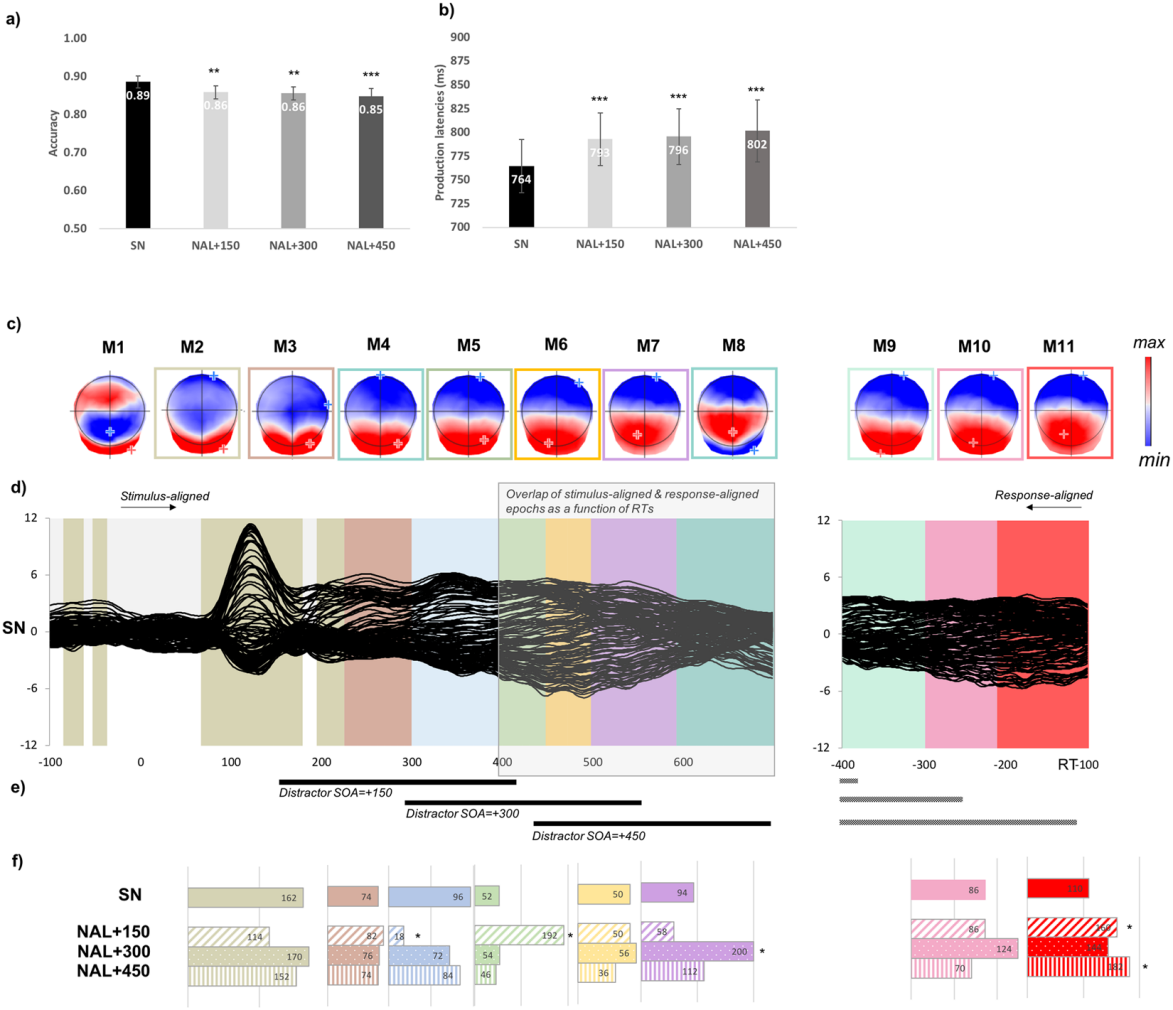
(**a,b**) Behavioral effects of listening to concurrent auditory syllables during picture naming. Accuracy and production latencies are presented for the single condition (SN) and in naming while listening to syllables at different SOAs (NAL + 150, NAL + 300 and NAL + 450). Grand-average ERPs (128 electrodes) for the picture naming single task from 100 ms prior to picture onset to 100 ms before the verbal response and temporal distribution of the microstates revealed by the spatio-temporal segmentation analysis. Microstates are color-coded (**d**). The white shaded area illustrates overlap between stimulus-aligned and response-aligned data as a function of mean production latency. Map templates for the eleven microstates observed from 100 ms prior to picture onset to RT-100 are also provided with positive (red) and negative (blue) values being displayed as well as maximal and minimal scalp field potentials (**c**). (**e**) Black lines illustrate the temporal profile of the appearance of distractors when aligned to stimuli. For information, gray dotted lines illustrate the same temporal profile when aligned to the response as a function of mean production latencies. (**f**) The histograms indicate the relative duration of each microstate and is given for each condition including the picture naming single condition displayed (SN) and the naming while listening to concurrent syllables with different SOAs: NAL + 150; NAL + 300 and NAL + 450. The actual duration of the microstates is displayed. *indicate significant differences (p < 0.05) observed on any specific microstate contrasting SN with each of the active interference conditions.

The analyses carried out on the active dual-task, in which the participants had to detect a target syllable (which was associated only to filler trials) are similar to those carried out on the passive hearing task. Behavioral data on 19 subjects revealed reduced accuracy and increased production latencies in picture naming with active listening to distractor syllables, no matter when the distractor appeared (see Fig. 5a,b). Significant differences in accuracy were found between the single condition (SN) and naming while actively listening when distractors appeared at +150 ms (89% vs. 86%; z = −2.65; p = 0.0082), at +300 ms (89% vs. 86%; z = −2.75; p = 0.006) or at +450 ms (89% vs. 85%; z = −3.28; p = 0.001). With regard to production latencies, a main effect of condition (F(3,3446) = 14.1, p < 0.001) and of stimuli order (F(1,3649) = 33, p < 0.001) were obtained. Increased production latencies in the active listening conditions relative to single naming were observed at SOA = +150 ms (29 ms slower than in SN, t(3467) = 4.96; p < 0.001), at SOA = +300 ms (32 ms slower, t(3438) = 4.64; p < 0.001) and at SOA = +450 ms (38 ms slower, t(3451) = 5.9; p < 0.001). No other significant differences were found (see Appendix B). Similar to what was observed in passive hearing, the results indicate that auditory syllables interfere with picture naming, even at a late SOA.

#### ERP results

In order to determine whether interference induced by actively listening to concurrent auditory syllables had the same locus as in passive hearing, microstate analyses were performed. A spatio-temporal segmentation analysis was run separately on the grand averaged stimulus-aligned (difference waves) and the response-aligned (raw) ERPs. The analysis yielded 8 different microstates on the stimulus-aligned data and 3 microstates on the response-aligned data in all conditions but with different time-distributions (see Fig. 5b–d). Despite slight differences in the distribution of microstates on stimulus-aligned ERPs compared to what was observed in passive hearing, the results indicate the same loci of interference i.e. on the last microstate close to verbal onset. Note that in this task, the effect is observed for SOAs = +150 ms and SOAs = +450 ms. Relative to single picture naming, a progressive shift in the microstates yielding longer duration across conditions is observed: M5 for picture naming with active listening to syllables presented at 150 ms relative to single picture naming (mean duration 192 ms vs. 52 ms, p < 0.001); M7 for picture naming with active listening to distractor syllables presented at 300 ms relative to single picture naming (mean duration 200 ms vs. 94 ms, p < 0.001) and M11 for picture naming with active listening to distractor syllables presented at 150 ms compared to single picture naming (mean duration 160 ms vs. 110; p = 0.031) and at 450 ms compared to single picture naming (mean duration 182 ms vs. 110 ms, p = 0.0062). Duration of M4 was reduced when syllables were displayed 150 ms after picture onset compared to single picture naming (mean duration 18 ms vs. 96 ms, p = 0.005).

### Direct across-task comparisons on production latencies

Results obtained in picture naming with passive hearing or active listening to auditory syllables seem similar although a gradual effect of interference was found in passive hearing. Across-tasks comparisons of production latencies were performed on 18 individuals (kept in EEG analyses in both tasks). Linear mixed-effects regression models were applied with Task (Active vs. Passive), Condition (single task, dual-task with SOA + 150, dual-task with SOA + 300 and dual-task with SOA + 450) and absolute stimuli order as fixed factors, and with participants and items as cross random effects. Significant effects of Task (F(1, 8060) = 34, p < 0.001), Condition (F(3, 8063) = 34, p < 0.001) and absolute stimuli order (F(1, 8173) = 958, p < 0.001) were obtained. The interaction Task*Condition was only marginal (F(3, 8099) = 2.59, p = 0.051), and was due to a marginal differential effect across tasks only at SoA + 150 (relative to single task: t(8102) = −1.7, p = 0.089).

## Discussion

Dual-task paradigms have been used to mimic everyday situations that lead people to get interfered, probably because of attentional limitations or of competing neural resources. Most available studies on this issue in the context of speaking are concerned with active dual-tasks i.e. when responses to two or more tasks are required. It is unclear however whether interference in active dual-tasks is only related to divided attention or also to competing resources when the two tasks involve overlapping modalities.

Here, we used a passive interference paradigm and an active paradigm as control experiment with the underlying assumption that passive hearing unrelated syllables attracts less attention than active listening. Participants heard verbal auditory stimuli while they planned single words and we analysed the mutual interference between utterance planning and hearing. Passively hearing concurrent auditory stimuli interfered with word planning, as indicated by increased production latencies for picture naming while ignoring auditory syllables, relative to when picture naming was achieved in isolation. Interference was observed at all stimulus onset asynchronies, even at the latest, i.e. when the auditory stimuli appeared 450 ms after picture onset. The analysis of ERP waveforms and microstates of picture naming revealed that this interference corresponded to modulations appearing ~200 ms before the vocal response.

In the reverse analysis on hearing (i.e. event-related activities locked to the onset of the auditory stimuli) a significant shift of latency of the N1 component of the auditory evoked potentials (AEP) was observed only when the syllables were presented at the latest SOA (+450 ms) although differences in amplitudes of both N1 and P2 were obtained at all SOAs.

Similar interference was observed when the task implied a higher degree of attentional load. Indeed, when participants actively listened to concurrent auditory stimuli, interference was obtained no matter the stimulus asynchrony. The locus of the interference is similar as in passive hearing i.e. on late time-windows close to the vocal response.

In the following, we will discuss the implications of these results for the comprehension of why and how utterance planning processes are interfered by concurrent auditory verbal stimuli even when they do not have to be attended by the speaker.

### Speech planning processes interfered while hearing

The present results in picture naming with passive hearing showing behavioral interference at late SOAs and modulation of ERPs in the time-window preceding articulation converge towards a post-lexical interference of auditory verbal stimuli on utterance planning. As for the earlier SOAs, increased duration of specific microstates was also obtained on stimulus-aligned epochs which, given their topographic configurations and temporal appearance, might just as well reflect post-lexical modulations. At first, these results support the idea that utterance form encoding may also be interfered, adding both behavioral and ERP evidence to previous similar claims^2,25^. Klaus *et al*. (2017) reported interference of a concurrent verbal memory task on the amount of phonological ahead planning of subject-verb-object sentences. Here, interference on post-lexical processes was observed regardless of the amount of attentional load, that is, when participants actively listened to or passively heard auditory syllables. Note though, our results may seem incompatible with previous observations from chronometric studies using the auditory picture word interference paradigm^26^. In such studies auditory verbal stimuli bearing different semantic or phonological relationships with the target words are used and compared to infer on word planning processes. For conditions of unrelated words it has been shown that production latencies were longer at early positive SOAs relative to silence^26^ (see introduction). Although such experimental manipulation served as baseline conditions for following experiments, results illustrate that auditory processing of unrelated words interfere with simultaneous picture naming. In subsequent picture-word interference studies however unrelated words were used as the baseline condition for related words and therefore not compared to silence and to our knowledge there are no other studies comparing unrelated verbal auditory stimuli to silence at different SOAs during picture naming. It is therefore possible that auditory stimuli delivered simultaneously with the to-be-named pictures (SOA = 0) would interfere with all subsequent processing stages. By contrast, when stimuli are presented with longer SOAs (e.g. +300 ms or +450 ms) lexical processes are likely to be completed, thus leaving only post-lexical processes to be potentially affected by concurrent auditory processing. If we follow this reasoning for the present experiment, both behavioral and ERPs effects suggest interference at late SOAs (>150 ms) hence that post-lexical processes (close to vocal onset) are affected by concurrent auditory processing. Note that we obtained similar interference in picture naming with active listening to syllables relative to picture naming in isolation, with similar effects across SOAs. These results replicate a previous study showing interference of post-lexical processes with SOA = +300 ms^25^, as well as a more recent study in aphasic participants^40^. In that study, aphasic participants showed increased phonological errors in dual-tasks with concurrent auditory stimuli presented at a late SOA (SOA = +300 ms) compared to picture naming in isolation. Finally, production latencies of healthy age-matched control participants showed the same dual-task interference as previously reported in young participants. Different effects as a function of SOAs across studies are thus likely to be due to the experimental designs such as the attention required in the tasks and baseline conditions (i.e. picture naming in isolation), but effects obtained on positive SOAs in several studies suggest that post-lexical processes are also interfered.

### Modulation of hearing while speaking

The analysis focusing on activities following auditory distractor onset indicated a shift of latency of the auditory component in the N1 time-window (around 100 ms) between passive hearing alone and hearing while planning words for speaking, but only when the syllables are heard at the latest word planning periods (SOA + 450 ms). Differences on amplitudes of auditory components were also seen. This indicates backward interference on low-level auditory processing while planning an utterance even during a task that does not require to actively respond to auditory stimuli. Modulations of AEP amplitudes or latencies have previously been reported in *active* dual-task conditions where they have been associated to central bottleneck and resource limitations^8,41–45^. For instance, Singhal and colleagues (2002) found reduced N1 amplitude to auditory targets in dual-task settings compared to when the task was completed in isolation^46^. In addition, Kasper *et al*.^10^ found latency shifts of the N1 in dual-tasks, correlating with RTs delays, which were taken to suggest that early processing of auditory stimuli was disrupted when attention was split across two tasks^47–49^. The backward interference varied according to the moment in the time-course of picture naming the auditory stimuli were presented, which argues in favor of interference originating not only from attention but also from competing neural resources. Daliri & Max^50^ recently reported increased latencies of the N1 component and amplitude modulations of the P2 component to syllables during simultaneous speech production (during articulation). In that case, modulations of auditory responses likely corresponded to the on-line influence of the central nervous system on the auditory system to reinforce its role in auditory feedback during overt speech articulation^46^. This cannot be the case here however as analyses were restricted to periods preceding vocal onset.

### Attentional load and competing resources between speech planning and auditory processes

Until now dual-task effects were mostly interpreted as resulting from limitations of attentional resources in line with single vs. multiple resources models^47,51–54^. Post-lexical processes are assumed to include word form (phonological and phonetic) encoding and monitoring of the planned utterance. Utterance form encoding precisely requires retrieving the segmental content (i.e. phonemes and syllables) of the words to be uttered, while supervision of the planning processes occurring prior to articulation (internal monitoring) is thought to occur on the encoded abstract phonological form^16,55–57^. There are two non-exclusive possible explanations for post-lexical interference. First, although instructions were to *ignore* distractors, passive hearing nevertheless attracts a sufficient amount of attention that is initially devoted to word-form encoding or monitoring processes, thus yielding to attentional interference. This first interpretation is supported by the similar interference observed when passive hearing and active listening dual-tasks are analysed jointly. Note that stimulus onset asynchronies of concurrent auditory syllables were random within an experimental block. It may thus be possible that participants could not ignore distractors as much as when they appear always at the same time. Such design is somehow closer to what happens in real life where concurrent stimuli can occur at any time and interfere with speech planning. Second, no matter the amount of attention required in the task, processing auditory syllables and encoding the phonological code for production may result in competing neural resources, thus contributing to the observed interference. Data in support of this idea are mixed. Increased interference in dual-tasks with verbal distractors (i.e. syllables) compared to non-verbal distractors (i.e. tones), have been previously reported in healthy individuals^25,34,40^ but verbal and non-verbal distractors yield to similar interference in left-hemisphere damaged participants^40^. Other studies showed influence of word planning on speech recognition when both share some phonological properties^58,59^. These studies showed notably that phonological processes engaged during active speech planning were also called for in speech recognition tasks such as phoneme monitoring or lexical decision, which were facilitated in case of shared phonemes. Further investigations are however required on this issue, in particular to explore and disentangle the respective influence of attentional demand and competing neural resources in dual-task interference.

Considering all previous studies showing that early lexical stages are affected in dual-tasks experiments, and recent work including the present study, there is now evidence that all speech planning processes, from conceptual retrieval to word-form encoding, are subject to “dual-task” interference.

## Conclusion

In the present study, we were able to show mutual modulations of speech planning processes during concurrent speech-related auditory processing, and of auditory processing during naming. Similar observations were made in two tasks requiring a differential amount of attention (naming pictures while passively hearing or actively listening to syllables) and results indicate that post-lexical processes of utterance planning are interfered by dual-task. In conversations, turn-taking seems usually smooth as next speakers start speaking right after the end of a previous turn or as soon as the incoming message has been understood^60,61^ which suggests that they start planning speech while still listening (or at least hearing) to the ongoing turn. However, other evidence show that listeners do not start planning their own turn-talk utterance as soon as they can anticipate the message of the speaker and rather wait until almost the end of the previous speech turn^62^, which may be related to the cost of planning while still hearing. In our study there was interference between planning and hearing although there was no phonological overlap between heard syllables and the pictures’ corresponding names. Hence to what extent such interference is enhanced or reduced in real conversation as a function of the overlap between the incoming message and the next planned utterance remains to be specified. Probably, people are always interfered to some extent, but this does not explicitly manifest in the behaviour, which is the result of a balance between anticipatory and monitoring mechanisms. Finally, these results don’t necessarily mean that multiple tasks cannot be performed in parallel. Listening to radio programs while driving or speaking on a phone for instance does not impair one’s performance but reducing the sound of the radio when speaking or writing may prevent cross-talk interference and/or lowering of attentional resources.

## Methods

### Participants

24 undergraduate students (4 men) took part in the study. All were native French speakers (mean age = 21.8 ± 4.2 years) and right-handed as determined by the Edinburgh Handedness Scale^63^. None of the participants had a history of psychiatric or neurological disorders and all had normal or corrected-to-normal vision. They gave their informed written consent in accordance with the declaration of Helsinki (1968) and got credit courses for their participation. Procedures were approved by the local faculty ethics committee of the University of Geneva (FPSE, University of Geneva, Geneva, Switzerland).

### Stimuli

In total, 323 pictures (line drawings on white squares) and their corresponding words were used in the experiment. 272 served as target pictures and the remaining 51 served as filler items. All pictures were repeated twice, once in the experimental block of passive hearing which is the purpose of the present study and once in another block of active task. The filler items were used as go trials in the active task which had a go/no-go design and were also presented in the passive hearing task presented here. Pictures depicted objects/entities and were selected from two French databases^64,65^ and all had high name agreement (>75%). Pictures were divided into four matched lists of items across condition: the picture naming without auditory distractors (single naming condition (SN)), and the three naming with passive hearing tasks (NPH) with various stimulus onset asynchronies (i.e.+150 ms; +300 ms and + 450 ms). Pictures and their corresponding names were matched across lists on several relevant psycholinguistic variables including name agreement, image agreement, familiarity, subjective visual complexity, image variability and age-of-acquisition (from the mentioned databases), lexical frequency, phonological neighborhood density, length (number of syllables and phonemes) (from Lexique^66^, but also on sonority of the first phoneme on an 8-points French sonority scale (see Appendix A). The four sets of pictures were counter-balanced across SOAs.

Five different syllables (targets: /Ri/, /na/, /mi/, /de/ fillers: /fo/) were selected as auditory stimuli, so that there was no phonological overlap between the distractor syllable and the first syllable of the target word. The duration of auditory syllables was 280 ms. There were 68 target trials per condition. Lists of items and order of blocks were counterbalanced across subjects.

### Procedure

Prior to the picture naming task, the participants underwent another task consisting in passive hearing the five auditory syllables (auditory single task or passive hearing PH) with the same number of trials as in the naming while hearing task. This was performed in a different block and consistently before the picture naming task throughout participants. Then, the participants underwent a picture naming task with and without concurrent auditory stimuli.

They were tested individually in a soundproof dark room. The presentation of trials was controlled by the E-Prime software (E-Studio). Pictures were presented in constant size of 240 × 245 pixels (about 4.52° of visual angle) on a black screen (approximately 60 cm from their chest). In the auditory single task, an experimental trial began with a fixation cross presented for 500 ms. Then the syllable was delivered through loudspeakers while a gray screen that was presented for 2000 ms. The participants did not have to attend particularly to the auditory stimuli. An inter-stimulus blank screen (1000 ms) was displayed afterwards. The passive hearing task was always presented before the naming while passive hearing but the order of passive and active tasks was counter-balanced across participants.

In the picture naming tasks, an experimental trial began with a fixation cross presented for 500 ms. Then the picture appeared on the screen for 2000 ms. In ¾ of the trials, an auditory syllable appeared either 150 ms, 300 ms or 450 ms after the onset of the picture (passive hearing). In the remaining ¼ of the trials, no auditory syllable was displayed (single naming condition, SN). Participants were requested to produce overtly the word corresponding to the picture as fast and accurate as possible, and ignore the concurrent auditory syllable when there was one. A blank screen lasting 1000 ms was displayed before the next trial. All four conditions were mixed up in a single block with a total of 68 target trials per condition.

In the active listening task, the experimental procedure was the same except that participants had to actively listen to unrelated distractors and detect specific syllables (/fo/) by pressing on a button box as fast as possible while concurrently producing overtly the word corresponding to the picture. These trials were filler trials and only no go trials were considered for further analyses.

### Behavioral analyses

Verbal responses were digitized with a microphone and systematically checked with a speech analysis software (CheckVocal 2.2.6^67^ to identify correct responses and production latencies (time between picture onset and vocal onset). No-responses, wrong responses (i.e. the participant produced a different name than the one expected), hesitations and/or auto-corrections during articulation were considered errors. Behavioral data were analyzed by means of mixed-effects regression models^68–70^ performed with the statistical software R (R Development Core Team, 2007) and the package lme4^71^ and lmerTest package, version 2.0-29^72^ using Satterthwaite approximation for degrees of freedom for the F statistics.

### EEG Acquisition and Pre-analyses

EEG was recorded continuously using the Active-Two Biosemi EEG system (Biosemi V.O.F. Amsterdam, The Netherlands) with 128 electrodes covering the entire scalp. Signals were sampled at 512 Hz (filters: DC to 104 Hz, 3 dB/octave slope). EEG activity was analyzed using the Cartool software^73^.

For the picture naming tasks, stimulus-aligned (800 ms) and response-aligned epochs (300 ms) were averaged for each task. Stimulus-aligned epochs started 100 ms before the onset of the picture to 700 ms post picture onset whereas response-aligned epochs were locked to the individual production latency of each trial (100 ms before vocal onset). For the auditory task, stimulus-aligned epochs (300 ms) starting at syllable onset to 300 ms afterwards were obtained.

Epochs in which amplitudes exceeded ±100 μV were rejected. In addition to this automated criterion, each trial was visually inspected and epochs contaminated by eye blinking, movement artefacts or other noise were excluded. Artifact electrodes were interpolated using 3-D splines interpolation^74^. Only trials corresponding to correct production and for which both stimulus-aligned and response-aligned epochs were available were retained for averaging (mean number of epochs = 51). ERPs were bandpass-filtered to 0.2–30 Hz (2^nd^ order acausal Butterworth filter with *−*12 dB/octave roll-off) with a notch filter (50 Hz) and were recalculated against the average reference.

To remove auditory evoked potentials due the auditory distractors for the ‘naming during passive hearing’ analysis, waveform differences were computed by subtracting the AEP elicited by syllables in isolation from the naming while hearing ERP (see below). This was obtained by extracting the AEP of the passive hearing task aligned to the onset of the syllable and by adding a pre-stimulus null signal which duration (150 ms, 300 ms, 450 ms) corresponded to the three SOA conditions.

For the ‘hearing while naming’ analysis (see below), ERPs from the single picture naming condition (SN) were subtracted from the ERPs in the three naming while hearing conditions on stimulus-aligned epochs only. After subtraction, ERPs were re-aligned to the onset of the respective auditory distractor (150, 300 and 450 ms).

#### Naming during passive hearing

The first ERP analysis was conducted on stimulus-aligned waveform differences calculated by subtracting the ERPs obtained in the passive hearing to auditory syllables (PH performed in a different block) from the picture naming while hearing tasks (NPH). This was done for each SOA by adding to the auditory ERPs to be subtracted a pre-stimulus null signal which duration was dependent on the SOA. This allowed us to remove auditory-related components observed in the picture naming tasks with concomitant auditory syllables. Subtractions were achieved under the additive model assumption which has been applied multiple times in the multisensory integration literature to compare multimodal and unimodal conditions^75–77^. The subtracted ERPs (NPH-PH) were then used to contrast with single picture naming condition (SN) performed in the same block as picture naming with passive hearing. Analyses were also performed on raw response-aligned epochs.

**Waveform analyses**: For this first analysis of waveforms, parametric ANOVAs were computed on amplitudes of the difference waves at each electrode and time point (2 ms) over the stimulus-aligned period and on raw waveforms on response-aligned period. Contrasts were performed only against the single task (i.e. SN vs. NPH + 150; SN vs. NPH + 300; SN vs. NPH + 450). To correct for multiple comparisons, a spatio-temporal clustering criterion was used: only differences observed over at least 5 adjacent electrodes and extending over at least 20 ms were retained with a conservative alpha criterion of 0.01.

Statistical analyses were performed using the STEN toolbox developed by Jean-François Knebel (http://www.unil.ch/line/home/menuinst/ianbout-the-line/software–analysis-tools.html).

**Microstates analysis**: The second analysis was a microstates analysis. This method allows summarizing ERP data into a limited number of stable topographic map configurations or microstates^78^. This method is independent from the used reference electrode^79,80^ and not sensitive to pure amplitude modulations across conditions: topographies of normalized maps are compared. The signal undergoes a segmentation that was obtained with the software Ragu^81^. The optimal number of microstates that best explained the group-averaged datasets was determined using the following procedure which was applied 50 times: Each time randomly splitting the 20 subjects into training and test datasets of 10 subjects each and testing between 1 and 20 microstates classes (for stimulus-aligned epochs; between 1 and 10 for response-aligned epochs). Every microstate identification run used the atomize and agglomerate hierarchical clustering method. Statistical smoothing was used to eliminate temporally isolated maps with low strength (10 points smoothing with a penalty factor of 3). The number of microstates with the higher correlation value is retained as the optimal number of maps for the current dataset. The spatio-temporal segmentation was applied to the grand-average data corresponding to the four conditions: single naming (SN), picture naming with auditory distractors syllables presented at 150 ms (NPH + 150), picture naming with auditory distractors syllables presented at 300 ms (NPH + 300) and picture naming with auditory distractors syllables presented at 450 ms (NPH + 450). The statistical validation of this analysis was tested thanks to a microstate fitting procedure during which each time point is labeled according to the map with which it best correlated spatially. This is achieved through randomization procedures such that for each participant, the ERPs corresponding to the conditions being compared are randomly assigned to arbitrarily defined groups. ERPs in these different groups are averaged and the variables of interest are computed (here map duration and offset) for the various conditions. After many repetitions, the variables of interest observed in the test dataset are compared with the empirical distributions under the null hypothesis. Details of this procedure can also be found in^82^.

#### Hearing while naming

In this analysis, waveforms differences were calculated by subtracting the ERPs of the single picture naming condition (SN) from the picture naming while hearing to syllables at each SOA (NPH-SN). This allowed us to obtain ‘auditory-evoked’ potentials in passive hearing tasks with removal of visual-related activity due to the picture. This was used to contrast with the passive hearing of syllables (PH) performed in isolation in a different block.

The global field power (GFP) was used to determine and extract auditory component latencies and amplitudes^83^ in single task and passive hearing tasks. As the N1 and P2 components peaked around 110 ms and 200 ms respectively, time-windows for further analyses were chosen accordingly: N1 time-window (75–150 ms after distractor onset) and P2 time-window (160–250 ms after distractor onset), in line with previous work^48^. Statistical comparisons were applied between PH and passive hearing at each SOA (NPH + 150, NPH + 300, NPH + 450) using Dunnett’s test for comparisons against a control condition and an alpha criterion of 0.05.

## Supplementary information

Supplementary material

## Data Availability

The datasets generated during and/or analysed during the current study are available from the corresponding author on reasonable request.
