# Supplementary material for: Interference in speaking while hearing and vice versa
Source: Sci Rep. 2019 Mar 29;9:5375. doi: 10.1038/s41598-019-41752-7 (PMC6441014; doi:10.1038/s41598-019-41752-7)
Supplement: Supplementary file 1 — Supplementary material [file 41598_2019_41752_MOESM1_ESM.docx]

**Interference in speaking while hearing and vice versa**

Raphaël Fargier^1,2^* & Marina Laganaro^2^

1. Aix-Marseille University, Institute of Language, Communication and Brain, Aix-en-Provence, France.

2. Faculty of Psychology and Educational Sciences, University of Geneva, Switzerland.

***Corresponding author:**

Raphaël Fargier

Mail: raphael.fargier@univ-amu.fr

Aix-Marseille University, ILCB

LPL

5 avenue Pasteur

13100 Aix-en-Provence

France

Appendix A: Table with psycholinguistic properties of counterbalanced lists of items.

|  | NA | IA | FAM | VC | IV | AoA | Freq | NbSyll | NbPho | PhoNeigh | Son |
| --- | --- | --- | --- | --- | --- | --- | --- | --- | --- | --- | --- |
| List1 | 94.3 | 3.7 | 3.2 | 2.9 | 2.9 | 2.2 | 39.4 | 1.7 | 4.3 | 9.1 | 3.3 |
| List2 | 94.6 | 3.6 | 3.1 | 3.0 | 3.0 | 2.1 | 43.5 | 1.6 | 3.9 | 10.3 | 3.5 |
| List3 | 94.8 | 3.7 | 3.0 | 2.8 | 3.0 | 2.1 | 46.0 | 1.8 | 4.4 | 8.5 | 3.1 |
| List4 | 93.0 | 3.8 | 3.2 | 2.8 | 2.8 | 2.2 | 28.8 | 1.7 | 4.3 | 10.0 | 3.1 |
|  |  |  |  |  |  |  |  |  |  |  |  |
|  | F=1.43, p=0.234 | F(3,201) = 1.39, p=0.24 | F<1 | F=1.061, p=0.367 | 1.541, p=0.21 | F=1.005, p=0.39 | F<1 | F<1 | F=1.488; p=0.219 | F<1 | F<1 |

NA=Name agreement, IA=Image agreement, FAM=Familiarity, VC= subjective Visual complexity, AoA=Age-of-acquisition (from picture databases); Freq=lexical frequency, NbSyll=length in syllables, NbPho=length in phonemes, PhoNeigh=Phonological neighbourhood density (from Lexique, New et al., 2004); Son=1^st^ phoneme sonority

Appendix B: Table with all statistical comparisons for behavioural results in the passive hearing task.

|  | **Accuracy** | | |  | **Production latencies** | | |
| --- | --- | --- | --- | --- | --- | --- | --- |
|  | *NPH+150* | *NPH+300* | *NPH+450* |  | *NPH+150* | *NPH+300* | *NPH+450* |
| *SN* | z=-1.37; *p*=0.172 | z=-1.6; *p*=0.11 | z=-3.39; *p*<0.001*** |  | t(4276)=3.596; *p*<0.001*** | t(4266)=4.66; *p*<0.001*** | t(4262) = 7.64; *p*<0.001*** |
| *NPH+150* |  | z=0.233; *p*=0.82 | z=2.05; *p*=0.04* |  |  | t(4256)=-1.05; *p*=0.296 | t(4264)=-4.048; *p*<0.001*** |
| *NPH+300* |  |  | z=1.82; *p*=0.07 |  |  |  | t(4270)=- 3.003; *p*=0.0027** |

The results of generalized and linear mixed-effects regression models for accuracy and production latencies respectively are given for each two-by-two comparisons. *p* values of t-tests against PH are provided. ****p*<0.001 ***p*<0.01 **p*<0.05

Appendix C: Table with analyses on GFP maxima latency and amplitude in ‘hearing while naming’

|  | **N1** | | | |  | **P2** | | | |
| --- | --- | --- | --- | --- | --- | --- | --- | --- | --- |
|  | Latency | | Amplitude | |  | Latency | | Amplitude | |
|  | *value* | *test against PH* | *value* | *test against PH* |  | *value* | *test against PH* | *value* | *test against PH* |
| **PH** | 110 |  | 1.99 |  |  | 192 |  | 2.4 |  |
| **PH+150** | 110 | 0.99 | 2.49 | 0.1 |  | 196 | 0.84 | 2.81 | 0.24 |
| **PH+300** | 116 | 0.80 | 2.5 | 0.09 |  | 202 | 0.42 | 2.98 | 0.06 |
| **PH+450** | 126 | 0.05* | 2.79 | 0.004** |  | 208 | 0.08 | 2.74 | 0.39 |

Latency and amplitudes were computed in the 75-150 ms time-window for the N1 and in the 160-250 ms time-window for the P2. *p* values from Dunnett’s test against PH are provided. ****p*<0.001 ***p*<0.01 **p*<0.05

Appendix D: Table with all statistical comparisons for behavioural results in the active listening task.

|  | **Accuracy** | | |  | **Production latencies** | | |
| --- | --- | --- | --- | --- | --- | --- | --- |
|  | *NAL+150* | *NAL+300* | *NAL+450* |  | *NAL+150* | *NAL+300* | *NAL+450* |
| *SN* | z=-2.64; *p=0.0083*** | z=-2.76; *p=0.006*** | z= -3.3; *p=*0.001*** |  | t(3467)=4.96; *p*<0.001*** | t(3438)= 4.64; *p*<0.001*** | t(3451) = 5.9; *p*<0.001*** |
| *NAL+150* |  | z<1 | z<1 |  |  | t(3477)<1 | t(3449)<1 |
| *NAL+300* |  |  | z<1 |  |  |  | t(3459)= 1.2; *p*=0.23 |

The results of generalized and linear mixed-effects regression models for accuracy and production latencies respectively are given for each two-by-two comparison.
